# High remnant cholesterol as a risk factor for developing chronic kidney disease in patients with prediabetes and type 2 diabetes: a cross-sectional study of a US population

**DOI:** 10.1007/s00592-024-02249-6

**Published:** 2024-03-04

**Authors:** Wenting Zhu, Qiushi Liu, Fang Liu, Chenfeng Jiao, Lihua Zhang, Honglang Xie

**Affiliations:** 1 National Clinical Research Center of Kidney Diseases, Jinling Hospital, Nanjing Medical Univerisity, Nanjing, 210016 China; 2 The Department of Urology, The First Affiliated Hospital of Anhui Medical University, Hefei, China; 3 Institute of Urology and Anhui Province Key Laboratory of Genitourinary Diseases, Anhui Medical University, Hefei, China

**Keywords:** Remnant cholesterol, Prediabetes, Type 2 diabetes mellitus, Chronic kidney disease, NHANES

## Abstract

**Aims:**

To examine any potential links between remnant cholesterol (RC) and comorbid chronic kidney disease (CKD) in individuals with prediabetes and type 2 diabetes mellitus (T2DM).

**Methods:**

We used data from 2709 American people aged > 20 years from the National Health and Nutrition Examination Survey (NHANES) during 2011–2018. Subjects were categorized according to whether they had comorbid CKD. Logistic regression models and smoothed curve fitting methods were employed to assess the association of RC with comorbid CKD in patients with prediabetes and T2DM.

**Results:**

The 2709 participants included 1473 patients with T2DM and 1236 with prediabetes [impaired glucose tolerance (IGT) and impaired fasting glucose (IFG)], of whom 744 (27.46%) had comorbid CKD. In multivariate-adjusted analysis, both RC and triglycerides (TG) were significantly associated with an increased risk of comorbid CKD, and a 1 mmol/L elevation of RC increased the risk by 38.1% [OR (95% CI) 1.636 (1.242, 2.156)], which was higher than the risk associated with a 1 mmol/L increase in TG [1.255 (1.106, 1.424)]. Additionally, those in the highest quartile of RC had a 43.6% higher risk of concomitant renal damage than those in the lowest quartile. RC was linearly and positively associated with the incidence of comorbid CKD in this population.

**Conclusions:**

RC is an independent risk factor for comorbid CKD in patients with prediabetes and T2DM. This finding provides a novel insight into the management and early detection of renal disease in patients with impaired glucose metabolism.

## Introduction

Chronic kidney disease (CKD) is becoming more common worldwide [1]. With a prevalence rate of between 30 and 40% in the USA, diabetes continues to be the primary cause of CKD in the majority of nations [2]. Nondiabetic hyperglycemia, which encompasses impaired fasting glucose (IFG) and impaired glucose tolerance (IGT), affects at least one-third of adults in the United States and one-fifth of adults in Europe [3]. Recent surveys have revealed that up to 30% of adults already demonstrate some degree of renal impairment at the time of their diabetes diagnosis [4]. Moreover, pathological evidence [5] suggests that the impact of hyperglycemia on the kidneys could have been initiated before blood glucose levels surpassed the threshold for diabetes [6]. Delaying CKD screening until after the onset of diabetes creates a missed opportunity for early prevention in many patients, which could result in significant national and global public health and economic consequences [7].

Similar to type 2 diabetes mellitus, prediabetes is linked to an atherogenic lipid profile and increased susceptibility to atherosclerotic cardiovascular disease (CVD) [8]. Hypertriglyceridemia decreased high-density lipoprotein cholesterol, and the appearance of small, dense low-density lipoprotein particles are the hallmarks of dyslipidemia in both conditions [9]. It has been established that remnant cholesterol (RC), a triglyceride-rich lipoprotein made up of very low-density lipoprotein, medium-density lipoproteins, and residual chylomicron particles, is positively correlated with the onset of diabetes and cardiovascular outcomes [10]. In the adult Chinese population, Zheng et al. discovered that elevated RC levels were positively associated with an increased risk of diabetes [11]. Numerous epidemiological, biological, and genetic investigations have confirmed the high atherogenic effect of RC in the type 2 diabetes population [12] and a strong correlation of RC with cardiovascular endpoints [13, 14]. Some systematic evaluation studies have revealed an association between diabetic microangiopathy and common macrovascular complications [15]. Several recent studies have reported that RC is directly linked to the onset of diabetic nephropathy [16] and can predict how the condition will progress [17]. High remnant cholesterol is a potential risk factor for the development of retinopathy in patients with type 2 diabetes [18]. Nonetheless, it remains uncertain whether RC constitutes a perilous element for the genesis of nephropathy among individuals with prediabetic conditions. In order to delve into this matter, we scrutinized information from the National Health and Nutrition Examination Survey (NHANES) to investigate the correlation between RC and CKD in patients with prediabetes and type 2 diabetes.

## Materials and methods

### Study population

**Fig. 1:**
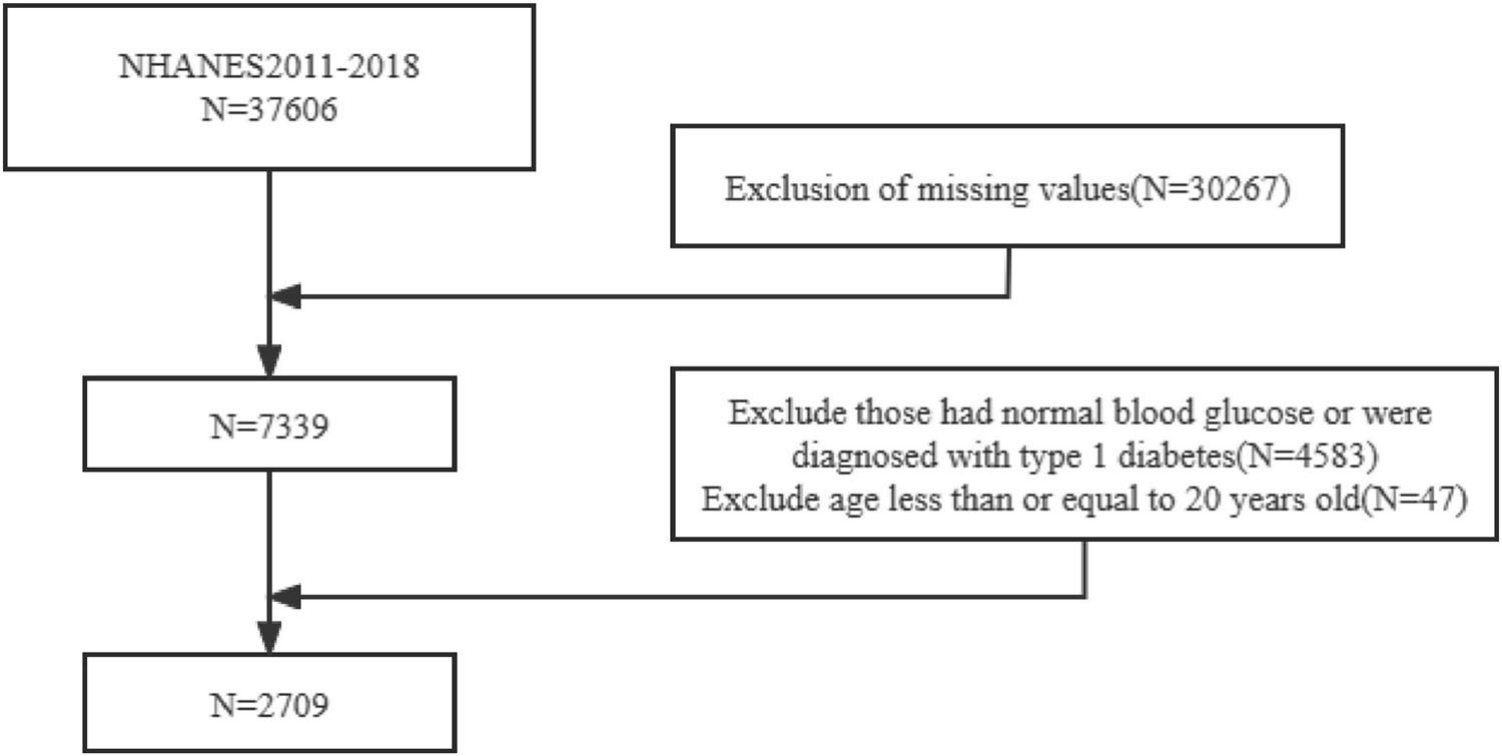
Flowchart of study participants

The NHANES survey data used in this research covered the years 2011 through 2018. To choose a qualified representative group of participants, NHANES used a complex multistage sampling process. The Centers for Disease Control and Prevention (CDC) conducts this poll every two years to monitor public health in the USA. The National Center for Health Statistics (NCHS) Institutional Review Board examined and approved the NHANES study plan before it was carried out, and all participants gave written informed consent. Our research examined data from four consecutive two-year survey cycles involving a total of 37,606 participants who had undergone a thorough health examination and a home interview. Subsequently, individuals exhibiting specific characteristics were excluded from the study, including 30,267 participants lacking lipid profiles or hematological data, 4583 participants diagnosed with type 1 diabetes or with normal blood glucose (NG), and 47 participants under the age of 20. Ultimately, our final analysis included data from 2709 participants, as illustrated in Fig. 1. All procedures were carried out following the necessary rules and regulations.

### Covariates, blood sample collection, and definition

The trained personnel gathered pertinent information on the study participants, including their age, sex, body mass index (BMI), race (Mexican American, non-Hispanic white, non-Hispanic black, and other races), poverty-to-income ratio (PIR), education level (less than high school, high school graduate, college or higher), smoking status (never, now, former) [19], alcohol consumption (never, moderate, heavy) [20], medical history, and medication use. A SBP of 140 mmHg or a DBP of 90 mmHg, the use of any anti-hypertensive medication, or a self-reported history of hypertension were all considered to be hypertension [21].

All venous blood samples were collected following a 9-h fasting period and were properly stored and transported at a temperature of  − 20℃. To determine fasting blood glucose (FBG) and glycosylated hemoglobin A1C (HbA1c), lipid profiles, including total cholesterol (TC), triglycerides (TG), high-density lipoprotein cholesterol (HDL-C), and low-density lipoprotein cholesterol (LDL-C), total protein, albumin (Alb), and routine blood markers including neutrophil count, lymphocyte count, and platelet count, the samples were analyzed in the University of Minnesota Fairview Medical Center laboratory employing the NHANES laboratory protocol [22].In addition, the first morning urine of each participant was collected and analyzed to calculate the urinary albumin creatinine ratio (ACR). RC was calculated in this study as TC minus LDL minus HDL [13]. By dividing the neutrophil count by the lymphocyte count, the neutrophil to lymphocyte ratio (NLR) values were calculated [23]. On the website wwwn.cdc.gov/Nchs/Nhanes/, specifics of the study variables used are available to the public.

As the guidelines set by the American Diabetes Association (ADA), we categorized the subjects as prediabetic if their fasting glucose levels were between 5.6 and 6.9 mmol/l (100–125 mg/dl, referred to as impaired fasting glucose; IFG), and/or their 2-h glucose levels ranged from 7.8 to 11.0 mmol/l (140–199 mg/dl, known as impaired glucose tolerance; IGT) [24]. The American Diabetes Association defined the diagnosis of diabetes as having a fasting glucose level above 7.0 mmol/L, an HbA1c level above 6.5%, the use of any antidiabetic medication, or a self-reported history of diabetes [25]. The serum creatinine equation developed by the Chronic Kidney Disease Epidemiology Collaborative was used to determine the estimated glomerular filtration rate (eGFR). An ACR of ≥ 30 mg/g and/or an eGFR of < 60 ml/min/1.73 m^2^ were used to define CKD [26].

### Statistical methods

For categorical variables, percentages were used to characterize the baseline characteristics of the study participants. Normally distributed data were described using means and standard deviations, while nonnormally distributed variables were described using medians and interquartile ranges. Significant differences between groups were assessed using nonparametric tests, one-way ANOVA, or Chi-square tests as appropriate. Before analysis, all covariates were screened for VIF values greater than 5, and any identified covariates were removed due to their collinearity. The study included three logistic regression models, which varied in terms of the covariates adjusted for. Specifically, Model 2 analyzed age, sex, and race, while Model 3 considered age, sex, race, education, PIR, smoking, alcohol, hypertension, fasting glucose, HbA1c, albumin, neutrophil count, lymphocyte count, platelet count, NLR, and lipid-lowering medication use. Additionally, a multivariate regression model was used to explore the relationship between lipid profile indicators and comorbid CKD by including RC quartiles as continuous variables. The link between these variables was then depicted using generalized additive model regression and smoothed curve fitting (penalized spline approach). Finally, the study used logistic regression models and log-likelihood ratio tests for interaction terms to examine whether there was heterogeneity in the connections between subgroups. The statistical significance level was set at *p* < 0.05.

Empower software (www.empowerstats.com; X&Y Solutions, Inc., Boston, MA, USA) and R version 3.4.3 (http://www.r-project.org, The R Foundation) were used for all analyses.

## Results

The study ultimately included 2709 participants, including 1473 with type 2 diabetes and 1236 with prediabetes (IGT and IFG), of whom 744 (27.46%) were also found to have comorbid CKD. The median age of this population was 60 (47–69) years, and 1457 (53.78%) were male; their baseline characteristics are summarized in Table 1. Subjects with comorbid CKD had significantly higher RC levels than those without CKD [0.62 (0.44–0.87) vs. 0.58 (0.41–0.83), *p* = 0.009]. Additionally, age, race, education, smoking, alcohol consumption, hypertension, lipid-lowering medication use, albumin, TG, TC, LDL-C, fasting blood glucose, HbA1C, neutrophil count, lymphocyte count, platelet count, and NLR were significantly different between the 2 subgroups of patients who were grouped according to whether they had CKD.

**Table 1:** Baseline characteristics of the study participants

Variables	All (*n* = 2709)	Non-CKD (* n* = 1965)	CKD (*n *= 744)	*p* value
Age, years	60.00 (47.00–69.00)	56.00 (44.00–66.00)	68.00 (58.00–77.00)	< 0.001
BMI, kg/m2	30.10 (26.20–35.00)	29.90 (26.20–34.70)	30.50 (26.40–35.70)	0.102
Total protein, /L	71.00 (68.00–75.00)	71.00 (69.00–74.00)	71.00 (68.00–75.00)	0.999
Albumin, g/L	42.00 (40.00–44.00)	42.00 (40.00–44.00)	41.00 (39.00–43.00)	< 0.001
TG, mmol/L	1.30 (0.90–1.84)	1.26 (0.88–1.81)	1.35 (0.96–1.90)	0.007
TC, mmol/L	4.84 (4.11–5.56)	4.89 (4.16–5.56)	4.63 (3.98–5.53)	0.003
HDL-C, mmol/L	1.24 (1.06–1.53)	1.27 (1.06–1.53)	1.22 (1.03–1.53)	0.152
LDL-C, mmol/L	2.82 (2.20–3.46)	2.87 (2.25–3.49)	2.69 (2.04–3.39)	< 0.001
RC, mmol/L	0.59 (0.41–0.85)	0.58 (0.41–0.83)	0.62 (0.44–0.87)	0.009
Fasting glucose, mmol/L	6.44 (5.94–7.44)	6.38 (5.94–7.22)	6.66 (6.04–8.60)	< 0.001
HbA1c (%)	6.00 (5.60–6.70)	5.90 (5.50–6.50)	6.25 (5.80–7.40)	< 0.001
Lymphocyte count, ×10^9^/L	2.00 (1.60–2.40)	2.00 (1.60–2.50)	1.80 (1.50–2.40)	< 0.001
Platelet count, ×10^9^/L	226.00 (189.00–270.00)	227.00 (192.00–272.00)	220.00 (182.00–264.25)	0.001
Neutrophil count, × 10^9^/L	4.00 (3.20–5.00)	3.90 (3.10–4.90)	4.30 (3.40–5.60)	< 0.001
NLR	2.00 (1.51–2.71)	1.95 (1.46–2.56)	2.33 (1.67–3.16)	< 0.001
Men, * n* (%)	1457 (53.78%)	1062 (54.05%)	395 (53.09%)	0.657
Race, *n* (%)				0.006
White	1031 (38.06%)	713 (36.28%)	318 (42.74%)	
Black	542 (20.01%)	390 (19.85%)	152 (20.43%)	
Mexicans	439 (16.21%)	331 (16.84%)	108 (14.52%)	
Others	697 (25.73%)	531 (27.02%)	166 (22.31%)	
PIR, *n* (%)				0.133
≤ 1	599 (22.11%)	420 (21.37%)	179 (24.06%)	
> 1	2110 (77.89%)	1545 (78.63%)	565 (75.94%)	
Education,* n* (%)				0.002
Less than high school	711 (26.25%)	498 (25.34%)	213 (28.63%)	
High school	639 (23.59%)	440 (22.39%)	199 (26.75%)	
More than high school	1359 (50.17%)	1027 (52.26%)	332 (44.62%)	
Smoke, *n* (%)				< 0.001
Never	1425 (52.60%)	1072 (54.55%)	353 (47.45%)	
Now	471 (17.39%)	346 (17.61%)	125 (16.80%)	
Former	813 (30.01%)	547 (27.84%)	266 (35.75%)	
Alcohol, *n* (%)				< 0.001
Never	927 (34.22%)	625 (31.81%)	302 (40.59%)	
Moderate	1318 (48.65%)	982 (49.97%)	336 (45.16%)	
Heavy	464 (17.13%)	358 (18.22%)	106 (14.25%)	
Hypertension,* n* (%)				< 0.001
No	1032 (38.10%)	883 (44.94%)	149 (20.03%)	
Yes	1677 (61.90%)	1082 (55.06%)	595 (79.97%)	
Lipid-lowering, *n* (%)				< 0.001
No	681 (25.14%)	586 (29.82%)	95 (12.77%)	
Yes	2028 (74.86%)	1379 (70.18%)	649 (87.23%)	
Diabetes category, *n* (%)				< 0.001
T2DM	1473 (54.37%)	954 (48.55%)	519 (69.76%)	
Prediabetes	1236 (45.63%)	1011 (51.45%)	225 (30.24%)	

Abbreviations: *BMI* body mass index, *TG* triacylglycerol, *TC* total cholesterol, *LDL-C* low-density lipoprotein cholesterol, *HDL-C* high-density lipoprotein cholesterol, *RC* remnant cholesterol, *HbA1c* glycosylated hemoglobin, *NLR* neutrophil-to-lymphocyte ratio, *PIR* poverty-to-income ratio

**Fig. 2:**
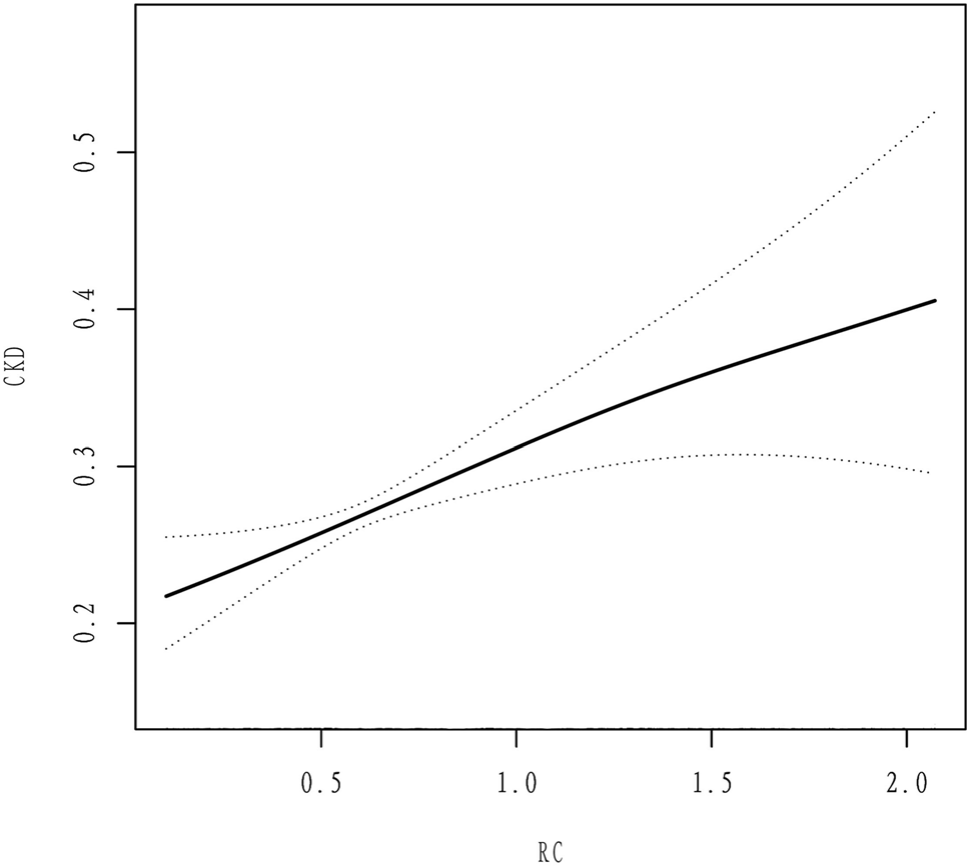
Density dose–response relationship between remnant cholesterol and comorbid CKD. *Notes* The area between two dotted lines is expressed as a 95% CI

In a comprehensively adjusted continuous analysis, both RC and TG exhibited a statistically significant association with an elevated risk of CKD. The OR (95% CI) values for RC and TG were 1.636 (1.242, 2.156) and 1.255 (1.106, 1.424), respectively. Furthermore, the risk of developing CKD for a 1 mmol/L increase in RC was 38.1% higher compared to a corresponding increase in TG. Notably, individuals belonging to the highest quartile of RC had a 43.6% higher risk of developing CKD than those in the lowest quartile of RC (Table 2). We further investigated the dose‒response association between RC and the risk of CKD using a generalized additive model and smoothed curve fitting. The findings revealed that RC was linearly and favorably related to the incidence of CKD (Fig. 2).

**Table 2:** OR (95% CI) of lipid profiles for risk of CKD

Exposure	Model1	Model2	Model3
OR (95% CI)			
RC Continuous, mmol/L	1.278 (1.017, 1.606) 0.036	1.727 (1.341, 2.222) < 0.001	1.636 (1.242, 2.156) < 0.001
RC Categorical, mmol/L			
≤ 0.41	1.0	1.0	1.0
> 0.41, ≤ 0.59	1.162 (0.908, 1.487) 0.233	1.204 (0.926, 1.565) 0.166	1.115 (0.844, 1.473) 0.442
> 0.59, ≤ 0.85	1.300 (1.020, 1.657) 0.034	1.375 (1.059, 1.785) 0.017	1.238 (0.936, 1.637) 0.134
> 0.85	1.320 (1.032, 1.687) 0.027	1.710 (1.307, 2.236) < 0.001	1.551 (1.157, 2.079) 0.003
P for trend	0.017	< 0.001	0.002
TG Continuous, mmol/L	1.121 (1.010, 1.245) 0.032	1.287 (1.146, 1.445) < 0.001	1.255 (1.106, 1.424) < 0.001
TG Categorical, mmol/L			
≤ 0.90	1.0	1.0	1.0
> 0.90, ≤ 1.30	1.155 (0.906, 1.474) 0.245	1.209 (0.933, 1.566) 0.151	1.155 (0.878, 1.520) 0.303
> 1.30, ≤ 1.84	1.292 (1.012, 1.649) 0.040	1.400 (1.077, 1.821) 0.012	1.282 (0.968, 1.697) 0.083
> 1.84	1.329 (1.043, 1.695) 0.022	1.731 (1.327, 2.259) < 0.001	1.591 (1.191, 2.126) 0.002
P for trend	0.014	< 0.001	0.001
TC Continuous, mmol/L	0.922 (0.853, 0.997) 0.041	0.997 (0.918, 1.083) 0.944	1.076 (0.985, 1.174) 0.103
TC Categorical, mmol/L			
≤ 4.11	1.0	1.0	1.0
> 4.11, ≤ 4.84	0.943 (0.750, 1.185) 0.615	1.076 (0.843, 1.374) 0.557	1.169 (0.904, 1.512) 0.233
> 4.84, ≤ 5.56	0.576 (0.450, 0.738) < 0.001	0.678 (0.522, 0.882) 0.004	0.864 (0.655, 1.140) 0.302
> 5.56	0.817 (0.646, 1.033) 0.092	1.030 (0.799, 1.327) 0.821	1.267 (0.965, 1.664) 0.089
P for trend	0.004	0.378	0.338
HDL.C Continuous, mmol/L	0.969 (0.782, 1.199) 0.771	0.644 (0.504, 0.824) < 0.001	0.831 (0.641, 1.078) 0.164
HDL.C Categorical, mmol/L			
< = 1.06	1.0	1.0	1.0
> 1.06, ≤ 1.24	1.013 (0.800, 1.282) 0.915	0.919 (0.714, 1.184) 0.515	0.986 (0.757, 1.284) 0.915
> 1.24, ≤ 1.53	0.797 (0.630, 1.008) 0.058	0.634 (0.491, 0.818) < 0.001	0.720 (0.550, 0.942) 0.017
> 1.53	0.983 (0.778, 1.242) 0.887	0.620 (0.475, 0.807) < 0.001	0.790 (0.594, 1.049) 0.103
P for trend	0.428	< 0.001	0.026
LDL.C Continuous, mmol/L	0.872 (0.797, 0.954) 0.003	0.991 (0.902, 1.088) 0.847	1.060 (0.960, 1.170) 0.251
LDL.C Categorical, mmol/L			
≤ 2.20	1.0	1.0	1.0
> 2.20, ≤ 2.82	0.821 (0.652, 1.034) 0.093	0.988 (0.773, 1.262) 0.922	1.147 (0.887, 1.484) 0.296
> 2.82, ≤ 3.46	0.588 (0.461, 0.750) < 0.001	0.753 (0.582, 0.975) 0.031	0.848 (0.646, 1.113) 0.236
> 3.46	0.702 (0.557, 0.886) 0.003	0.964 (0.752, 1.237) 0.776	1.175 (0.901, 1.534) 0.234
P for trend	< 0.001	0.361	0.642

Model 1 adjusted for None

Model 2 adjusted for age, sex, and race

Model 3 adjusted for age, sex, race, education, PIR, smoking, alcohol, hypertension, fasting glucose, HbA1c, albumin, neutrophil count, lymphocyte count, platelet count, NLR, and lipid-lowering medication use

Abbreviations: *PIR* poverty-to-income ratio, *HbA1c* glycosylated hemoglobin, *NLR* neutrophil-to-lymphocyte ratio, *RC* remnant cholesterol, *TG* triacylglycerol, *TC* total cholesterol, *LDL-C* low-density lipoprotein cholesterol, *HDL-C*: high-density lipoprotein cholesterol

**Fig. 3:**
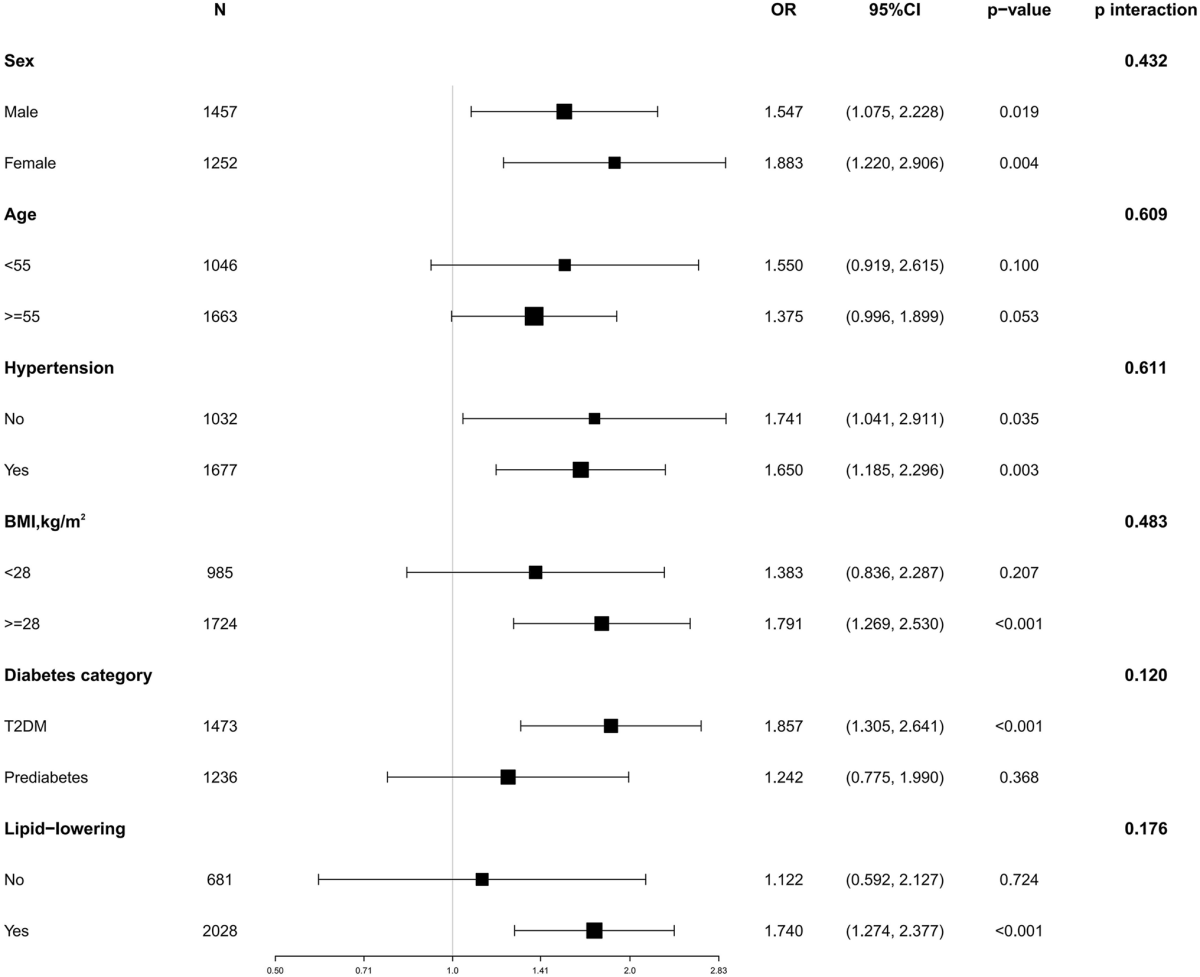
Odds ratios for the connection between RC and risk of CKD in various subgroups. *Notes* Theses odds ratios are derived from in logistic regression Model 3

During the subgroup analysis (Fig. 3), RC and risk of comorbid CKD did not interact significantly with age, sex, hypertension, BMI, lipid-lowering drug use, or type of diabetes (all interactions *p* > 0.05). This suggests that the association between RC and the risk of CKD was consistent across all six prespecified subgroups.

## Discussion

This cross-sectional study, conducted in the USA aimed to explore the relationship between RC and comorbid CKD in individuals with impaired glucose metabolism. A direct and positive association with a linear trend was observed between increased RC concentrations and renal damage. Additionally, the subgroup analysis confirmed the correlation between RC and renal damage. These results indicate that elevated RC levels represent a substantial risk factor for CKD, particularly in patients with prediabetes and type 2 diabetes. This helps us early detect and preventively manage kidney disease in patients with abnormal glucose metabolism.

Dyslipidemia is more common in people with impaired glucose metabolism than in people with normal blood glucose (NG) levels [27]. Even with the utilization of statins to lower LDL-C, diabetes patients still face a higher residual risk of CVD. Several studies suggest that the RC produced by very low-density lipoprotein and chylomicron lipolysis contributes to this residual risk [28]. A substantial correlation between CKD and CVD events has been demonstrated by numerous cross-sectional and prospective studies [29]. The development and progression of diabetic CKD may be influenced by dyslipidemia, which has been associated with lower eGFR and higher ACR in previous epidemiological and genetic studies [30]. Traditional lipid parameters such as TG have been considered among the indicators that show a strong correlation with diabetic nephropathy, which is consistent with our results [31]. Elevated RC levels were found as considerable proteinuria resulting from the progression of diabetic nephropathy, according to a study on 105 Japanese T2DM patients [32]. Wu et al. found that high RC was independently associated with the development of diabetic nephropathy [16]. Studies have shown that RC can predict the onset of both type 1 and type 2 diabetic retinopathy [17]. In addition, there is evidence that patients with prodromal DM are more prone to DM and have higher RC than patients with NG [33]. Hadi et al. reported that RC is linked to prediabetes, and they hypothesized that RC may affect glucose metabolism [34]. In the current investigation, we add to the evidence that RC is positively associated with combined renal damage in patients with impaired glucose metabolism.

Several factors have been identified as important drivers of atherosclerotic calcification in diabetes, including oxidative stress, altered mineral metabolism, endothelial dysfunction, increased production of inflammatory cytokines, and the release of bone progenitor cells from the bone marrow into circulation [35]. Given the partial concordance between the etiology of diabetic microvascular and macrovascular complications [36], it has been postulated that inflammation and insulin resistance may serve as potential mediators between RC and diabetic CKD. First, RC can be captured and absorbed by macrophages, where it can then be converted into foam cells and inflame nearby endothelial cells [12]. Elevated levels of IL-6 and CRP, indicators of inflammation, may reactivate adipocytes to a large extent. For the end site, IL-6 and downstream CRP induction may be associated with the corelease of other pathogenic subsites caused by other irritating adipocytes [37]. Varbo et al. discovered a causal link between RC and low-grade inflammation, even in the general population, unlike LDL-C [38]. Leukocyte count and albuminuria were found to be related in a recent study of people with type 2 diabetes [39]. Another study from China reported that leukocyte count, even within the normal range, was associated with diabetic microvascular complications [40]. Additionally, increased interleukin levels in inflammatory conditions can cause lymphocytopenia and neutrophilia, which can elevate the NLR [37]. Furthermore, in subjects with type 2 diabetes, the NLR has been reported to indicate the level of control of diabetes [23]. Second, insulin resistance is seen in the initial stages of diabetic CKD and has been linked to the development of endothelial dysfunction, oxidative stress, and mild inflammation [41], which are closely associated with glomerular hyperfiltration, proteinuria production, and decreased renal function [42]. To ensure the validity of our findings, we adjusted for the neutrophil count, lymphocyte count, platelet count, and NLR among blood indicators in a multifactorial regression model. Despite these observations, further investigations are needed to fully elucidate the precise mechanisms underlying the association between RC and diabetic CKD.

According to a NHANES report spanning from 2009 to 2012, it was estimated that 35.3% of US adults (equivalent to approximately 80.8 million individuals) had impaired fasting glucose [43]. Additionally, Melsom et al. conducted a study emphasizing the importance of targeting prediabetes for early treatment to prevent diabetic CKD [44]. Given that conventional treatment fails to reduce the risk of cardiovascular and renal disease associated with diabetic CKD, it is crucial to identify prediabetes as a potential disease entity, detect it at an early stage, and treat early renal abnormalities such as ultrafiltration and proteinuria in a timely fashion, thus stemming the tide of renal insufficiency. In Fig. 3 of this study, we performed subgroup analyses for type 2 diabetic and prediabetic subjects, and P for interaction showed that the association between RC and risk of comorbid CKD was consistent across the two populations of type 2 diabetic and prediabetic subjects, which confirms the robustness of the results. Therefore, early intervention for renal damage in pre-diabetic populations deserves our attention.

It should be noted that this study has certain limitations. First, due to its observational nature and cross-sectional design, establishing a causal relationship between RC levels and kidney damage was not feasible. Second, while the study utilized formula-calculated fasting RC to represent “residual” cholesterol levels, direct measurement was not conducted, and hence, the calculated residual cholesterol levels may have been relatively high, owing to the inclusion of cholesterol from newly formed VLDL particles. Nevertheless, the use of indirect calculation for RC is generally simple and convenient, making it commonplace in large-scale population studies. Third, it could have been inaccurate in the elderly given that HbA1c levels increase with age, potentially affecting red blood cell lifespan and, consequently, the prevalence of diabetes [45]. Finally, due to the limited nature of the data, we did not perform a detailed categorization of the types of lipid-lowering medications used to assess whether they were a potential biasing factor.

To summarize, elevated fasting levels of RC are an autonomous contributor to the emergence of CKD among individuals with prediabetes and type 2 diabetes. There is reason to foresee the monitoring of RC concentrations as a viable strategy to appraise and act upon patients who fall in the category of increased susceptibility to renal afflictions. This could slow the onset of intricate and hard-to-treat diabetic nephropathy. The management of lipid profiles in individuals undergoing impaired glucose metabolism warrants the development of protocols via randomized clinical trials with prolonged follow-up.

## Data Availability

The datasets analyzed during the current study are available on the NHANES official website, https://wwwn.cdc.gov/Nchs/Nhanes/.

## Abbreviations

CKD: Chronic kidney disease
T2DM: Type 2 diabetes mellitus
NHANES: National health and nutrition examination survey
IGT: Impaired glucose tolerance
IFG: Impaired fasting glucose
CVD: Cardiovascular disease
NG: Normal blood glucose
ACR: Albumin creatinine ratio
eGFR: Estimated glomerular filtration rate
BMI: Body mass index
TG: Triacylglycerol
TC: Total cholesterol
LDL-C: Low-density lipoprotein cholesterol
HDL-C: High-density lipoprotein cholesterol
RC: Remnant cholesterol
HbA1c: Glycosylated hemoglobin
NLR: Neutrophil-to-lymphocyte ratio
PIR: Poverty-to-income ratio
