# Correction to: High remnant cholesterol as a risk factor for developing chronic kidney disease in patients with prediabetes and type 2 diabetes: a cross-sectional study of a US population

**DOI:** 10.1007/s00592-024-02274-5

**Published:** 2024-04-16

**Authors:** Wenting Zhu, Qiushi Liu, Fang Liu, Chenfeng Jiao, Lihua Zhang, Honglang Xie

**Affiliations:** 1 National Clinical Research Center of Kidney Diseases, Jinling Hospital, Nanjing Medical Univerisity, Nanjing, 210016 China; 2 The Department of Urology, The First Affiliated Hospital of Anhui Medical University, Hefei, China; 3 Institute of Urology and Anhui Province Key Laboratory of Genitourinary Diseases, Anhui Medical University, Hefei, China

**Correction to: Acta Diabetologica** 10.1007/s00592-024-02249-6

In the sentence beginning ‘In multivariate-adjusted analysis, both RC and' in this article, "OR" was mislabeled as "HR".

In the sentence beginning ‘The OR (95% CI) values for RC and TG were 1.636’ "OR" was mislabeled as "HR".

The Table 2 caption was incorrectly given as ‘Hazard ratio (95% CI) of lipid profles for risk of CKD' but should have been 'Odds ratio (95% CI) of lipid profiles for risk of CKD'.

The original article has been corrected.

